# Controlled Growth of Porous InBr_3_: PbBr_2_ Film for Preparation of CsPbBr_3_ in Carbon-Based Planar Perovskite Solar Cells

**DOI:** 10.3390/nano11092408

**Published:** 2021-09-16

**Authors:** Kailin Chi, Hansi Xu, Bingtao Feng, Xianwei Meng, Daoyu Yu, Qian Li

**Affiliations:** 1 School of Science, Northeast Electric Power University, Jilin 132012, China; 20182815@neepu.edu.cn (H.X.); 2018309050138@neepu.edu.cn (D.Y.); 2 State Key Laboratory of Superhard Materials, College of Physics, Jilin University, Changchun 130012, China; fengbt20@mails.jlu.edu.cn (B.F.); xwmeng17@mails.jlu.edu.cn (X.M.); 3 Beijing Key Lab of Cryo-Biomedical Engineering and Key Lab of Cryogenics, Technical Institute of Physics and Chemistry, Chinese Academy of Sciences, Beijing 100190, China

**Keywords:** CsPbBr_3_, planar perovskite solar cells, InBr_3_, porous PbBr_2_ film

## Abstract

Due to the low solubility of CsBr in organic solvents, the CsPbBr_3_ film prepared by the multi-step method has holes and insufficient thickness, and the light absorption capacity and current density of the perovskite film hinder the further improvement in the power conversion efficiency (PCE) of CsPbBr_3_ solar cells. In this study, we introduced InBr_3_ into the PbBr_2_ precursor solution and adjusted the concentration of PbBr_2_, successfully prepared PbBr_2_ with a porous structure on the compact TiO_2_ (c-TiO_2_) substrate to ensure that it fully reacted with CsBr, and obtained the planar carbon-based CsPbBr_3_ solar cells with high-quality perovskite film. The results reveal that the porous PbBr_2_ structure and the increasing PbBr_2_ concentration are beneficial to increase the thickness of the CsPbBr_3_ films, optimize the surface morphology, and significantly enhance the light absorption capacity. Finally, the PCE of the CsPbBr_3_ solar cells obtained after conditions optimization was 5.76%.

## 1. Introduction

From the perspective of the preparation process, a very unique advantage of perovskite solar cells (PSCs) is that they have better processability [1,2,3,4]. Whether an organic-inorganic hybrid or all-inorganic perovskite solar cell, the film quality of the perovskite active layer (including morphology, grain shape, grain size, number of grain boundaries, whether there are pinholes, etc.) determines the PCE and durability of PSCs [5,6,7,8,9]. Generally, the prepared perovskite film should have a high-density microstructure, large grain size, low grain boundary density, and no pinholes or other voids in the film, to improve electron/hole mobility, achieve reduction in radiation recombination, and other purposes [1,10,11,12]. Both the preparation method of the perovskite film and the device structure (planar or mesoporous structure) can exert a significant impact on the quality and photoelectric performance of the perovskite film. In the process of preparing perovskite films by the two-step method, mesoporous substrates are often used to promote the PCE of PSCs. This is because the mesoporous layer formed by oxide nanoparticles has a higher specific surface area, and a large number of pores promote the improvement in the effective diffusivity of the lead halide precursor solution [13], so denser lead halide nanosized grains can be generated faster in the mesoporous layer, and the conversion process of lead halide to perovskite can be accelerated [1,14]. However, during this conversion process, due to some unconverted lead halide residues in the mesoporous layer and the possible formation of random crystals in the perovskite film [14,15], the efficiency of PSCs may be greatly affected. A method for fully converting the lead halide is a critical prerequisite for controlling the growth of perovskite films, or ensuring the perfect formation of crystals and enhancing the efficiency of PSCs [16,17,18].

Introducing a porous lead halide film into a planar structure is an effective method to replace the mesoporous structure in the two-step method [1]. The porous structure in the lead halide film can enlarge the contact area between the lead halide and other precursor solutions and increase the reaction rate, and it can reduce space-expansion-induced defects and residual stress during the expansion of the film [5]. Liu et al. [19] spin-coated a solution of PbI_2_ in DMF onto a substrate, then prepared a PbI_2_ film with a porous structure by adjusting the standing time. Ostwald ripening of the PbI_2_ crystallites can result in larger crystals and increase the void space between grains. The increase in porosity in the PbI_2_ film effectively promotes the transformation process of perovskite and reduces the residual amount of PbI_2_. The resulting CH_3_NH_3_PbI_3_ PSCs with inverted planar heterojunction have an efficiency of 15.7%, and there is almost no hysteresis. Dong et al. [20] confirmed that the porosity in PbI_2_ can affect the phase composition and film morphology of CsPbI_2_Br. Compared with other anti-solvents, the PbI_2_ (DMSO) film treated with green ethanol has higher porosity and randomly distributed crystals, which provide more diffusion paths and contact areas for the CsBr precursor, and create an essential prerequisite for ultimately obtaining CsPbI_2_Br film with high purity, high thickness, large grains, and full coverage. Although the CsPbBr_3_ film prepared by the multi-step method exhibits excellent moisture and thermal stability [21,22,23,24], it is also prone to problems such as low film coverage, thin film thickness, and the presence of impurity phases (such as CsPb_2_Br_5_) [5,25,26]. As mentioned above, a porous PbBr_2_ film with higher porosity can provide more diffusion paths for the CsBr precursor and increase the contact area between the two, which ultimately provides a favorable condition for the formation of CsPbBr_3_ with high purity phase and high coverage [27,28,29]. Zhao et al. [21] prepared porous PbBr_2_ films on SnO_2_ substrates by precisely controlling the crystallization temperature. The porous structure of PbBr_2_ not only allows the effective diffusion of CsBr solution to gain high-purity CsPbBr_3_, but also offers enough space for the growth of CsPbBr_3_ grains under stress-free conditions, resulting in high-quality CsPbBr_3_ film with larger grain size and lower grain boundary density. In our previous work, we prepared porous PbBr_2_ films on mesoporous TiO_2_ (m-TiO_2_) by introducing InBr_3_ into the PbBr_2_ precursor solution, and achieved In^3+^ or In cluster doping with CsPbBr_3_, thereby improving the growth quality of CsPbBr_3_ films and enhancing the efficiency of CsPbBr_3_ solar cells with mesoporous structure [30].

At present, it is a highly attractive choice to use carbon electrodes, which are low cost and highly stable, to construct CsPbBr_3_ PSCs without hole transport layer (HTL). Carbon, which matches the work function of perovskite, can effectively transport holes, and avoid the instability of traditional organic HTL (such as Spiro-MeOTAD) and the low conductivity of metal oxide HTL. At the same time, compared with Au electrodes, the carbon electrode is lower cost, and does not diffuse into the perovskite and cause the device performance to deteriorate as does the Ag electrode [31,32,33]. On the basis of our previous research results, in this work, we introduced InBr_3_ into a PbBr_2_ precursor solution and adjusted the concentration of PbBr_2_ to prepare a high-quality CsPbBr_3_ film with high thickness, high density, and no holes on c-TiO_2_, in order to further simplify the structure of the CsPbBr_3_ PSCs and optimize the growth quality of the CsPbBr_3_ film. When the concentration of InBr_3_ is 0.21 M and PbBr_2_ concentration is 1.3 M, the prepared carbon-based CsPbBr_3_ solar cells with a planar structure show the best 5.76% PCE with a short circuit current density (J_SC_) of 6.52 mA/cm^2^, an open circuit voltage (V_OC_) of 1.29 V, and a fill factor (FF) of 0.68.

## 2. Materials and Methods

### 2.1. Materials

The fluorine-doped tin oxide coated glass (FTO, 6 Ω/□) was purchased from Opvtech New Energy Co., Ltd. (Yingkou, China). Titanium (IV) isopropoxide (99.9%), N,N-dimethylformamide (DMF, chromatographic grade, ≥99.9%), methanol (chromatographic grade, ≥99.9%), ethanol (chromatographic grade, ≥99.8%), and isopropanol (≥99.5%) were purchased from Aladdin (Shanghai, China). PbBr_2_ (99.99%) and CsBr (99.9%) were purchased from Xi’an Polymer Light Technology Corp (Xi’an, China). InBr_3_ (99.9%) was purchased from Macklin Biochemical Co., Ltd. (Shanghai, China). The commercial carbon paste was purchased from Shanghai MaterWin New Materials Co., Ltd. (Shanghai, China).

### 2.2. Device Fabrication

All the following processes were carried out in an air atmosphere. The c-TiO_2_ was spin-coated on pretreated FTO substrates using 0.2 M titanium isopropoxide in ethanol at 5000 rpm for 30 s and dried for 10 min at 120 °C, then the substrates were annealed at 500 °C for 1 h. Perovskite films were synthesized by a multistep solution-processing method. We added 0.21 mmol of InBr_3_ to 1 mL DMF solution of 1.0, 1.1, 1.2, 1.3, or 1.4 M PbBr_2_, which was stirred under 90 °C. Afterward, the mixed solution was spin-coated on the FTO/c-TiO_2_ substrate at 2000 rpm for 30 s and heated to 90 °C for 30 min to obtain InBr_3_:PbBr_2_ films with a porous structure. Then, the methanol solution of CsBr (0.07 M) was spin-coated on InBr_3_:PbBr_2_ film at 5000 rpm for 30 s and continuingly heated at 250 °C for 5 min. This step was performed 6 times. After the prepared sample was soaked in isopropanol for 30 min and annealed at 250 °C for 15 min, the blade coating method was applied to coat carbon paste onto the perovskite films and heated at 100 °C for 10 min to form the carbon back electrode with the effective area of 0.09 cm^−2^.

### 2.3. Characterization

The X-ray diffraction (XRD) patterns of the synthesized sample were investigated using an X-ray diffractometer (Cu Kα radiation, λ = 1.5418 Å, Rigaku D/max2500, Tokyo, Japan). The morphologies of the synthesized films were recorded by a scanning electron microscope (SEM, FEI MAGELLAN 400, FEI, Hillsboro, OR, USA), and the system was connected to an energy-dispersive X-ray spectroscopy (EDS). X-ray photoelectron spectroscopy (XPS) was performed by an X-ray photoelectron spectrometer system (K-Alpha, Thermo Fisher Scientific, Waltham, MA, USA) equipped with a monochromatic Al Kα X-ray source (1486.6 eV) operating at 100 W. The steady-state photoluminescence (PL) and the time-resolved photoluminescence (TRPL) decay spectrums of perovskite films were collected on a photoluminescence spectrometer (FLS980, Edinburgh Instruments, Livingston, UK) with a 473 nm excitation source. The absorption spectrum was measured by a UV–Vis spectrometer (UV-3600, Shimadzu, TKY, Japan) in the range of 200 to 800 nm. The current–voltage (*J–V*) characteristics were measured by a solar cells test system (XP3000, Sanyou, China) equipped with a Keithley 2400 source meter. The external quantum efficiency (EQE) was tested by an EQE measured system (QTest Station 1000A, CROWNTECH, INC., Macungie, PA, USA) under DC mode.

## 3. Results and Discussion

**Figure 1:**
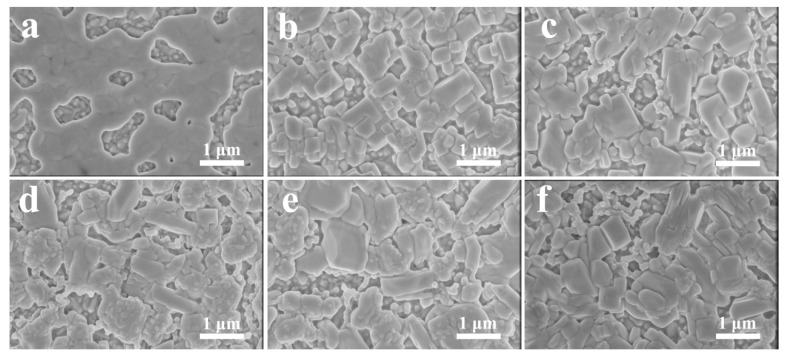
Top-view SEM images of PbBr_2_ films synthesized with different PbBr_2_ concentration: (**a**) 1.0 M w/o InBr_3_; (**b**) 1.0 M w/ InBr_3_; (**c**) 1.1 M w/ InBr_3_; (**d**) 1.2 M w/ InBr_3_; (**e**) 1.3 M w/ InBr_3_; (**f**) 1.4 M w/ InBr_3_.

Figure 1 displays the top-view SEM images prepared without and with introducing 0.21 M InBr_3_ in PbBr_2_ precursors of different solubility. When the PbBr_2_ precursor concentration was 1.0 M without introducing InBr_3_, the PbBr_2_ film produced was uniform, dense, crack-free, and had a smooth surface, and there were pores of different sizes in the film (Figure 1a). In contrast, after the introduction of InBr_3_ into the 1.0 M PbBr_2_ precursor solution, as shown in Figure 1b, the InBr_3_:CsPbBr_3_ film had a porous structure, the crystal grains were mostly block structures with obvious size differences, and there were relatively small gaps between the crystal grains. This indicates that InBr_3_ can transform the PbBr_2_ grown on the c-TiO_2_ substrate from a dense film to a relatively low-density porous film. Compared with the porous PbBr_2_ film formed on m-TiO_2_, the morphology of the PbBr_2_ film prepared on c-TiO_2_ changed significantly regardless of the introduction of InBr_3_, which reveals that both the TiO_2_ substrate and InBr_3_ can directly affect the morphology of the PbBr_2_ film and determine the quality of the subsequent CsPbBr_3_ film growth. With the further increase in the PbBr_2_ concentration (1.1~1.4 M), although the films prepared under various conditions were still porous films, the morphology and porosity of the films did not illustrate obvious differences. The columnar and cubic crystal grains grouped into clusters, with gaps between the clusters, which provide space for the subsequent growth and free expansion of CsPbBr_3_ crystals [13,34]. Based on the cross-sectional SEM images of the corresponding PbBr_2_ films (Figure S1, Supplementary Materials), it can be concluded that the thickness of the film (330, 400, 450, 500, and 550 nm), the grain size, and the gap between the crystal grains (clusters) all exhibit an increasing trend with the increase in the PbBr_2_ concentration, which provides the pivotal condition to enhance the diffusion rate of the CsBr precursor and increase the contact area and reaction efficiency between CsBr and PbBr_2_, and subsequently guarantee the production of high-quality CsPbBr_3_ films with high thickness, high purity, high density, and large crystal grains.

**Figure 2:**
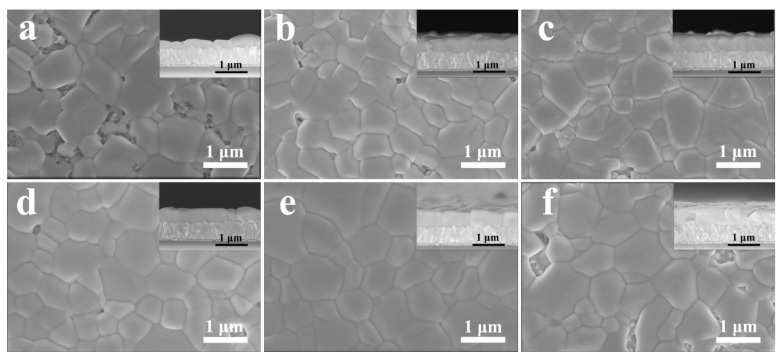
Top-view and cross-sectional (insets) SEM images of CsPbBr_3_ films synthesized with different PbBr_2_ concentration: (**a**) 1.0 M w/o InBr_3_; (**b**) 1.0 M w/ InBr_3_; (**c**) 1.1 M w/ InBr_3_; (**d**) 1.2 M w/ InBr_3_; (**e**) 1.3 M w/ InBr_3_; (**f**) 1.4 M w/ InBr_3_.

To validate the influence of porous lead bromide films made by introducing InBr_3_ on the morphologies of the CsPbBr_3_ films, the top-view and cross-sectional SEM images illustrated in Figure 2 were examined. When the PbBr_2_ precursor concentration was 1.0 M without introducing InBr_3_, that is, when the PbBr_2_ film was a flat film, the thickness of the obtained CsPbBr_3_ film was about 290 nm, the film showed poor uniformity and coverage, and many holes in the film (Figure 2a). With the further increase in PbBr_2_ concentration (1.1~1.4 M), the thickness of each perovskite film was 310, 330, 350, and 370 nm, respectively (Figure S2, Supplementary Material). When 0.21 M InBr_3_ was introduced into the PbBr_2_ precursor solution, with the increase in the PbBr_2_ concentration (1.0~1.3 M), the CsPbBr_3_ film showed a satisfying growth relationship with c-TiO_2_, and the film thickness significantly increased to approximately 430, 470, 500, and 540 nm, respectively, which reveals that the porous structure of the PbBr_2_ film is the main factor affecting the increase in the thickness of the CsPbBr_3_ film. Meanwhile, the coverage of the CsPbBr_3_ film gradually rose with the increase in the PbBr_2_ concentration, with the pores in the film gradually decreasing, the average size of the crystal grains increasing, and the density and uniformity of the perovskite film significantly improving. In particular, when the PbBr_2_ concentration was 1.3 M, the surface of the CsPbBr_3_ film was smooth and dense with almost no holes. As mentioned above, the porous structure of the PbBr_2_ film can increase the contact area between the CsBr precursor and PbBr_2_ and make the two fully react, thereby making the process of converting PbBr_2_ to CsPbBr_3_ faster and more complete. With the increase in the PbBr_2_ concentration, that is, as the gap between the PbBr_2_ crystal grain clusters gradually enlarges, more uniform perovskite nuclei with a narrower size distribution are generated in the porous PbBr_2_ film in a shorter time. Small grains with unfavorable growth orientation transfer their monomers through the solid-state grain boundary to at least a few larger grains with decent growth orientation, until smaller grains disappear to generate large grains with the largest surface and interface area, and finally producing a large-grain CsPbBr_3_ film that completely covers the substrate and has no holes [1,35]. However, as the concentration of the PbBr_2_ precursor containing InBr_3_ was further increased to 1.4 M, the thickness of the formed CsPbBr_3_ film further increased to about 580 nm, but the surface morphology of the CsPbBr_3_ film began to deteriorate, and holes appeared in the film again, which can adversely affect the J_SC_ of CsPbBr_3_ solar cells [36].

**Figure 3:**
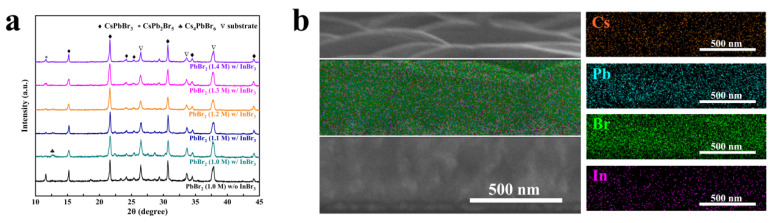
(**a**) XRD patterns of InBr_3_:CsPbBr_3_ films synthesized with different PbBr_2_ concentration; (**b**) elemental distribution mapping images of the InBr_3_:CsPbBr_3_ synthesized with the PbBr_2_ concentration of 1.3 M.

To investigate the effect of porous PbBr_2_ film on the phase, structure, and element composition of the perovskite, the XRD and EDS mappings were employed to characterize the CsPbBr_3_ films. The XRD patterns demonstrated in Figure 3a reveal that all CsPbBr_3_ films are cubic structures (PDF# 54-0752) [21,37], which reflects that the introduction of InBr_3_ and the concentration change of the PbBr_2_ film will not change the phase of CsPbBr_3_. When the PbBr_2_ precursor concentration was 1.0 M without introducing InBr_3_, a strong diffraction peak belonging to the PbBr_2_-rich CsPb_2_Br_5_ phase appeared at 2θ of 11.7°. In contrast, after introducing InBr_3_ into the 1.0 M PbBr_2_ precursor, the diffraction peak belonging to CsPb_2_Br_5_ phase disappeared (CsPb_2_Br_5_ will not be completely consumed), but the diffraction peak belonging to Cs_4_PbBr_6_ phase appeared at 2θ of 12.7°. This is because after the porous PbBr_2_ film with low thickness is fully contacted with the CsBr solution and is completely consumed, the excess CsBr and CsPbBr_3_ continue to react and form a CsBr-rich Cs_4_PbBr_6_ phase. On the contrary, in the planar structure of PbBr_2_, the contact area and the reaction efficiency between CsBr and PbBr_2_ are relatively low, and thus PbBr_2_ is not fully consumed. After the introduction of InBr_3_, as the concentration of the PbBr_2_ precursor gradually increased from 1.1 to 1.4 M, the intensity of the diffraction peaks belonging to the CsPb_2_Br_5_ phase in the XRD patterns of each sample showed an upward trend, and no obvious diffraction peak belonging to Cs_4_PbBr_6_ phase was observed. The cross-sectional EDS mapping of the CsPbBr_3_ film shown in Figure 3b proves that Cs, Pb, Br, and In are evenly distributed in the analyzed area, and there is no Pb or In gathering in a certain area.

**Figure 4:**
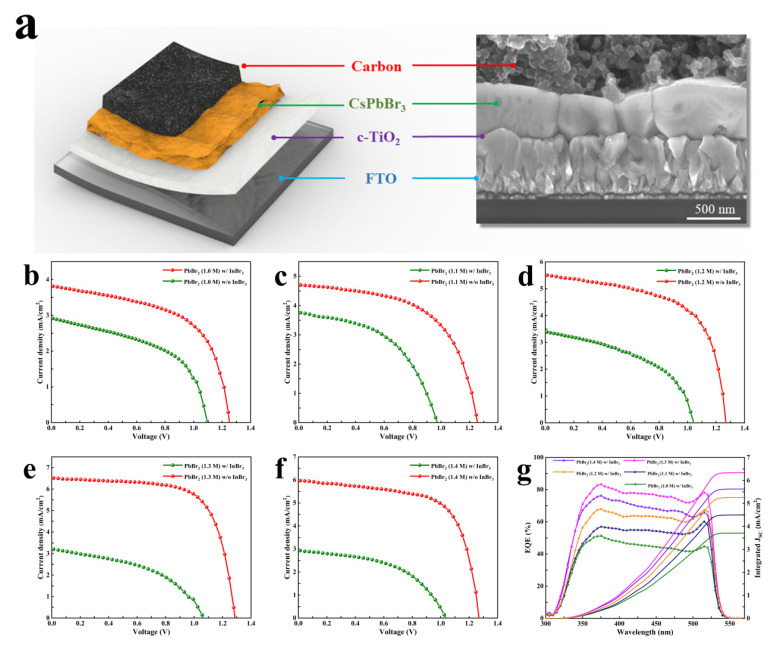
(**a**) Cross-sectional SEM image of the InBr_3_:CsPbBr_3_ device; (**b**–**f**) *J–V* characteristics and (**g**) EQE spectra and integrated photocurrent densities for the InBr_3_:CsPbBr_3_ devices synthesized with different PbBr_2_ concentrations.

Afterward, we coated the prepared CsPbBr_3_ films with commercial carbon paste, and dried them to obtain the planar HTL-free PSCs with the structure of FTO/c-TiO_2_/CsPbBr_3_/carbon; the cross-sectional SEM image of the device is shown in Figure 4a. In order to further explore the effect of PbBr_2_ porous film on the device performance, Figure 4b–f illustrates the *J–V* curves under reverse scanning of the PSCs prepared under the conditions of different concentrations of PbBr_2_ precursor solutions without and with introducing 0.21 M InBr_3_, and the corresponding parameters are summarized in Table 1. When the PbBr_2_ concentration was gradually increased from 1.0 to 1.4 M without introducing InBr_3_ into the precursor solution, the efficiency of each CsPbBr_3_ cell was at a relatively low level, and there was little difference in the results. It is noteworthy that the efficiency of the CsPbBr_3_ cell obtained under the condition of a PbBr_2_ concentration of 1.2 M was the highest, namely 1.79%, while the efficiency of the other cells was between 1.5% and 1.7%. After the introduction of InBr_3_ into the PbBr_2_ precursor solution, the efficiency of all PSCs was significantly improved. As the PbBr_2_ concentration increased from 1.0 to 1.3 M, J_SC_, V_OC_, FF, and PCE all showed a trend of increasing with the increase in PbBr_2_ concentration. When the PbBr_2_ concentration was 1.3 M, the champion device with a PCE of 5.76% showed a J_SC_ of 6.52 mA/cm^2^, a V_OC_ of 1.29 V, and an FF of 0.68. When the PbBr_2_ concentration was further increased to 1.4 M, the efficiency of the device decreased to 4.97%, and J_SC_, V_OC_, and FF also illustrated a slight decline, caused by the slight deterioration in the morphology of CsPbBr_3_. The statistics of photovoltaic parameters collected from 15 devices (Figure S3, Supplementary Materials) and the average photovoltaic parameters with a small standard deviation (Table S1, Supplementary Materials) demonstrate that the InBr_3_:CsPbBr_3_ PSCs synthesized with different PbBr_2_ concentrations obtained in this work have decent reliability and reproducibility. Figure 4g displays the EQE and the corresponding integrated photocurrent densities to verify the validity of the CsPbBr_3_ devices based on InBr_3_. The photoresponse edges of these five devices are all around 540 nm. When the PbBr_2_ concentration is 1.3 M and the maximum EQE value of the champion device is 83%, the EQE values in the light absorption wavelength range of 350–525 nm are significantly enhanced, which proves that the device has better charge injection ability and collection capacity. In addition, the integrated photocurrent density of each device is closer to the corresponding J_SC_, and the mismatch is less than 5%, which signifies the reliability of the *J–V* curves.

**Figure 5:**
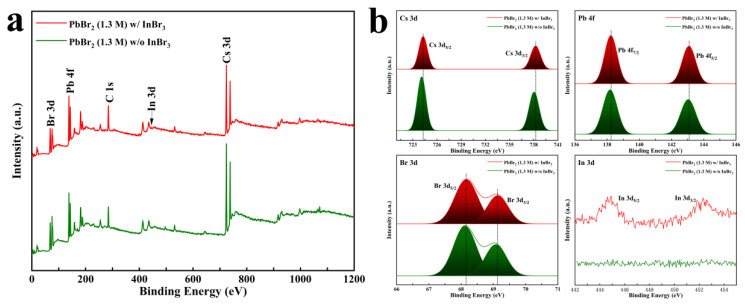
(**a**) XPS spectra and (**b**) Cs 3d, Pb 4f, Br 3d, and In 3d core spectra of the InBr_3_:CsPbBr_3_ synthesized with the PbBr_2_ concentration of 1.3 M.

Furthermore, XPS was utilized to study the influence of InBr_3_ on the electronic state of CsPbBr_3_ films at a PbBr_2_ concentration of 1.3 M. As displayed in Figure 5a, compared with the pure CsPbBr_3_, in addition to Cs 3d, Pb 4f, and Br 3d, In 3d was found in the XPS spectra for InBr_3_: CsPbBr_3_ film. According to Figure 5b, In 3d_5/2_ and In 3d_3/2_ peaked at 444.9 eV and 452.3 eV, respectively, and the high-resolution core-level binding energies of Cs 3d, Pb 4f, and Br 3d all shifted toward higher values compared with those in the pure CsPbBr_3_, which indicates that part of the In(III) in the porous PbBr_2_ was doped into the perovskite lattice in the process of transforming CsPbBr_3_, and caused the chemical state of [PbBr_6_]^4−^ octahedron to change [38]. The incorporation of In^3+^ or In cluster not only improves the spatial symmetry of the CsPbBr_3_ lattice structure, but also reduces vacancy defects, which is beneficial to the extraction and transfer process of the charge, and finally significantly optimizes the J_SC_ of the device [30,38,39,40].

**Figure 6:**
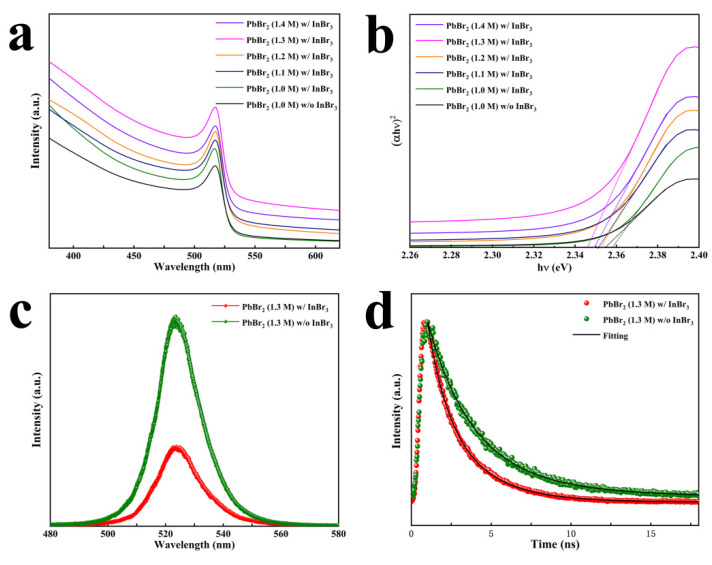
(**a**) UV–vis absorption spectra and (**b**) (αhν)^2^ vs. hν plots for the InBr_3_:CsPbBr_3_ devices synthesized with different PbBr_2_ concentrations; (**c**) PL and (**d**) TRPL decay spectra of InBr_3_:CsPbBr_3_ synthesized with the PbBr_2_ concentration of 1.3 M.

In the optical property test, the devices with the FTO/c-TiO_2_/CsPbBr_3_ structure were further characterized by a UV–visible spectrophotometer and PL. In Figure 6a, as expected, the absorption edges of all perovskite films are at around 533 nm, which is basically the same as the EQE test result. The light absorption capacity of the films shows a trend of first increasing and then decreasing with increasing PbBr_2_ concentration, which is consistent with the change law of the perovskite morphology and PCE. In other words, the increase in the thickness of the CsPbBr_3_ film and the optimization of the surface morphology can enhance the light absorption rate of the perovskite layer and reduce the light loss, which is conducive to generating more electrons to improve J_SC_. Using Tuac’s equation, we can calculate the corresponding band gap (E_g_) patterns of the CsPbBr_3_ film; the curves of α^2^ versus the photon energy hν, as shown in Figure 6b; and the E_g_ of the prepared CsPbBr_3_ to be about 2.35 eV. Regardless of whether InBr_3_ was introduced into the PbBr_2_ precursor, the solubility of PbBr_2_ did not meaningfully change the E_g_ of CsPbBr_3_. Figure S4 (Supplementary Material) illustrates the PL spectra of the PSCs prepared under different concentrations of PbBr_2_ precursor solutions with introducing 0.21 M InBr_3_. All the InBr_3_:CsPbBr_3_ films presented a typical emission band around 523 nm, which is close to their optical band gap. The PL intensity decreased as the PbBr_2_ concentration increased from 1.0 to 1.3 M, and then increased as the PbBr_2_ concentration further increased to 1.4 M. The InBr_3_:CsPbBr_3_ film synthesized with the PbBr_2_ concentration of 1.3 M showed the lowest PL intensity. When the PbBr_2_ precursor concentration was 1.3 M with/without introducing InBr_3_, as shown in Figure 6c, the PL peaks for both the CsPbBr_3_ films were still at about 523 nm, which indicates that the change in the concentration of PbBr_2_ and the introduction of InBr_3_ do not change the band position. However, the perovskite prepared after the introduction of InBr_3_ into the PbBr_2_ precursor solution showed more observable strong quenching, which proves that the incorporation of In^3+^ or In cluster and the improvement of perovskite film growth quality are beneficial to faster electron injection and collection, and reduce the recombination of electrons and holes [41,42], which explains why the J_SC_ and V_OC_ of the PSCs were significantly improved after the introduction of InBr_3_ in Figure 4b–f. Figure 6d illustrates the TRPL decay curves, and the relevant detailed parameters of the corresponding CsPbBr_3_ films are listed in Table S2. The specific lifetime of the carriers can be determined by a bi-exponential formula $I=A_{1}e^{-\left(\tau -\tau_{0}\right)/\tau_{1}}+A_{2}e^{-\left(\tau -\tau_{0}\right)/\tau_{2}}$ (A_1_ and A_2_ are the relative amplitude, respectively; *τ*_1_ and *τ*_2_ are the fast and slow decay time, respectively) [43], and the average lifetimes (*τ*_ave_) is calculated by the formula ${\;\tau }_{\mathrm{ave}}=\left(A_{1}\tau_{1}^{2}+A_{2}\tau_{2}^{2}\right)/\left(A_{1}\tau_{1}+A_{2}\tau_{2}\right)$ [44]. Significantly decreased lifetimes of *τ*_1_, *τ*_2_, and *τ*_ave_ of the InBr_3_:CsPbBr_3_ film (1.35, 3.06 and 2.49 ns, respectively) compared with the control CsPbBr_3_ (2.65, 5.61, and 3.95 ns, respectively) were observed, representing lower trap states in the InBr_3_:CsPbBr_3_ film and an accelerated electron transport process. This phenomenon is brought about by the enhancement in the growth quality of InBr_3_:CsPbBr_3_ film and the reduction in the defects of Pb^2+^ and Br^−^ after partial substitution of Pb^2+^ by In^3+^ or In cluster [25,39]. This is conducive to improving the V_OC_ and fill factor (FF) of the devices [45].

## 4. Conclusions

By introducing InBr_3_ into a PbBr_2_ precursor solution, we successfully prepared planar carbon-based CsPbBr_3_ solar cells with higher perovskite film quality in this work. The introduction of InBr_3_ can transform the dense PbBr_2_ film originally grown on c-TiO_2_ into a porous structure. On this basis, appropriately increasing the PbBr_2_ concentration in the DMF precursor solution can enhance the thickness of the CsPbBr_3_ film and optimize the surface morphology without changing the phase of CsPbBr_3_, which can effectively alleviate the low thickness and poor quality of the CsPbBr_3_ film in the multi-step process. Due to the increase in film thickness and the incorporation of In^3+^ or In cluster, the light absorption capacity of the CsPbBr_3_ film is significantly improved. Therefore, the efficiency and EQE of the manufactured devices are significantly optimized. As InBr_3_ is introduced into the current flooding fluid and the PbBr_2_ concentration is 1.3 M, the small-area CsPbBr_3_ solar cell has the best PCE, which is 5.76%, and the maximum EQE value is 83%. This work provides a certain reference for the preparation of high-quality CsPbBr_3_ thin films, simplifying the structure and developing planar HTL-free CsPbBr_3_ solar cells.

## Figures and Tables

**Table 1:** Key *J-V* parameters of the corresponding CsPbBr_3_ solar cells.

CsPbBr_3_		J_SC_ (mA/cm^2^)	V_OC_ (V)	FF	PCE (%)
**1.0 M**	**w/o InBr_3_**	2.97	1.09	0.50	1.62
	**w/ InBr_3_**	3.82	1.25	0.57	2.72
**1.1 M**	**w/o InBr_3_**	3.47	0.97	0.49	1.79
	**w/ InBr_3_**	4.70	1.26	0.57	3.38
**1.2 M**	**w/o InBr_3_**	3.40	1.04	0.47	1.66
	**w/ InBr_3_**	5.51	1.27	0.60	4.20
**1.3 M**	**w/o InBr_3_**	3.22	1.07	0.46	1.58
	**w/ InBr_3_**	6.52	1.29	0.68	5.76
**1.4 M**	**w/o InBr_3_**	2.94	1.04	0.50	1.53
	**w/ InBr_3_**	5.97	1.27	0.65	4.97

## Data Availability

The data is available on reasonable request from the corresponding author.

## Supplementary Materials

The following are available online at https://www.mdpi.com/article/10.3390/nano11092408/s1, Figure S1: Cross-sectional SEM images of PbBr_2_ films synthesized with different PbBr_2_ concentration: (a) 1.0 M w/ InBr_3_; (b) 1.1 M w/ InBr_3_; (c) 1.2 M w/ InBr_3_; (d) 1.3 M w/ InBr_3_; (e) 1.4 M w/ InBr_3_; Figure S2: Cross-sectional SEM images of CsPbBr_3_ films synthesized with different PbBr_2_ concentration: (a) 1.1 M w/o InBr_3_; (b) 1.2 M w/o InBr_3_; (c) 1.3 M w/o InBr_3_; (d) 1.4 M w/o InBr_3_; Figure S3: The schematic diagram of of 15 CsPbBr_3_ PSCs synthesized with different PbBr_2_ concentration: (a) JSC, (b) VOC, (c) FF and (e) PCE. Table S1: The average photovoltaic parameters of 15 CsPbBr_3_ PSCs synthesized with different PbBr_2_ concentration; Figure S4: PL spectra of the InBr_3_:CsPbBr_3_ synthesized with different PbBr_2_ concentration; Table S2: The fitted data of TRPL characterization.

## References

[1] Dunlap-Shohl W., Zhou Y., Padture N.P., Mitzi D.B. (2019). Synthetic Approaches for Halide Perovskite Thin Films. Chem. Rev..

[2] Yuan J., Ling X., Yang D., Li F., Zhou S., Shi J., Qian Y., Hu J., Sun Y., Yang Y. (2018). Band-Aligned Polymeric Hole Transport Materials for Extremely Low Energy Loss α-CsPbI3 Perovskite Nanocrystal Solar Cells. Joule.

[3] Jeon I., Seo S., Sato Y., Delacou C., Anisimov A., Suenaga K., Kauppinen E.I., Maruyama S., Matsuo Y. (2017). Perovskite Solar Cells Using Carbon Nanotubes Both as Cathode and as Anode. J. Phys. Chem. C.

[4] Weber S., Rath T., Mangalam J., Kunert B., Coclite A.M., Bauch M., Dimopoulos T., Trimmel G. (2018). Investigation of NiOX-hole Transport Layers in Triple Cation Perovskite Solar Cells. J. Mater. Sci. Mater. Electron..

[5] Chen W., Li X., Li Y., Li Y. (2020). A review: Crystal Growth for High-Performance All-Inorganic Perovskite Solar Cells. Energy Envi-ron. Sci..

[6] Wang K., Liu C., Du P., Zhang H.-L., Gong X. (2015). Efficient Perovskite Hybrid Solar Cells Through a Homogeneous High-Quality Organolead Iodide Layer. Small.

[7] Yuan S., Qiu Z., Gao C., Zhang H., Jiang Y., Li C., Yu J., Cao B. (2016). High-Quality Perovskite Films Grown with a Fast Solvent-Assisted Molecule Inserting Strategy for Highly Efficient and Stable Solar Cells. ACS Appl. Mater. Interfaces.

[8] Hu H., Ren Z., Fong P.W., Qin M., Liu D., Lei D., Lu X., Li G. (2019). Room-Temperature Meniscus Coating of >20% Perovskite Solar Cells: A Film Formation Mechanism Investigation. Adv. Funct. Mater..

[9] Liu Z., Deng K., Zhu Y., Wang M., Li L. (2018). Iodine Induced PbI 2 Porous Morphology Manipulation for High-Performance Planar Perovskite Solar Cells. Sol. RRL.

[10] Aristidou N., Eames C., Sanchez-Molina I., Bu X., Kosco J., Islam M.S., Haque S.A. (2017). Fast oxygen diffusion and iodide defects mediate oxygen-induced degradation of perovskite solar cells. Nat. Commun..

[11] Yen H.-J., Liang P.-W., Chueh C.-C., Yang Z., Jen A.K.-Y., Wang H.-L. (2016). Large Grained Perovskite Solar Cells Derived from Single-Crystal Perovskite Powders with Enhanced Ambient Stability. ACS Appl. Mater. Interfaces.

[12] Swarnkar A., Marshall A.R., Sanehira E.M., Chernomordik B.D., Moore D.T., Christians J.A., Chakrabarti T., Luther J.M. (2016). Quantum dot-induced phase stabilization of -CsPbI3 perovskite for high-efficiency photovoltaics. Science.

[13] Zhou Y., Game O.S., Pang S., Padture N.P. (2015). Microstructures of Organometal Trihalide Perovskites for Solar Cells: Their Evolution from Solutions and Characterization. J. Phys. Chem. Lett..

[14] Ummadisingu A., Grätzel M. (2018). Revealing the detailed path of sequential deposition for metal halide perovskite formation. Sci. Adv..

[15] Scherer G.W. (2004). Stress from crystallization of salt. Cem. Concr. Res..

[16] Chen Q., Zhou H., Song T.-B., Luo S., Hong Z., Duan H.-S., Dou L., Liu Y., Yang Y. (2014). Controllable Self-Induced Passivation of Hybrid Lead Iodide Perovskites toward High Performance Solar Cells. Nano Lett..

[17] Carmona C.R., Gratia P., Zimmermann I., Grancini G., Gao P., Graetzel M., Nazeeruddin M.K. (2015). High efficiency methylammonium lead triiodide perovskite solar cells: The relevance of non-stoichiometric precursors. Energy Environ. Sci..

[18] Han Q., Ding J., Bai Y., Li T., Ma J.-Y., Chen Y.-X., Zhou Y., Liu J., Ge Q.-Q., Chen J. (2018). Carrier Dynamics Engineering for High-Performance Electron-Transport-Layer-free Perovskite Photovoltaics. Chem.

[19] Liu T., Hu Q., Wu J., Chen K., Zhao L., Liu F., Wang C., Lu H., Jia S., Russell T.P. (2016). Mesoporous PbI2 Scaffold for High-Performance Planar Heterojunction Perovskite Solar Cells. Adv. Energy Mater..

[20] Dong C., Han X., Li W., Qiu Q., Wang J. (2019). Anti-solvent assisted multi-step deposition for efficient and stable carbon-based CsPbI2Br all-inorganic perovskite solar cell. Nano Energy.

[21] Zhao Y., Duan J., Wang Y., Yang X., Tang Q. (2020). Precise stress control of inorganic perovskite films for carbon-based solar cells with an ultrahigh voltage of 1.622 V. Nano Energy.

[22] Tian J., Xue Q., Yao Q., Li N., Brabec C.J., Yip H. (2020). Inorganic Halide Perovskite Solar Cells: Progress and Challenges. Adv. Energy Mater..

[23] Kulbak M., Gupta S., Kedem N., Levine I., Bendikov T., Hodes G., Cahen D. (2016). Cesium Enhances Long-Term Stability of Lead Bromide Perovskite-Based Solar Cells. J. Phys. Chem. Lett..

[24] Zhang J., Hodes G., Jin Z., Liu F. (2019). All-Inorganic CsPbX 3 Perovskite Solar Cells: Progress and Prospects. Angew. Chem. Int. Ed..

[25] Subhani W.S., Wang K., Du M., Wang X., Liu F. (2019). Interface-Modification-Induced Gradient Energy Band for Highly Efficient CsPbIBr 2 Perovskite Solar Cells. Adv. Energy Mater..

[26] Duan J., Zhao Y., He B., Tang Q. (2018). High-Purity Inorganic Perovskite Films for Solar Cells with 9.72% Efficiency. Angew. Chem. Int. Ed..

[27] Sun K., Hu Z., Shen B., Lu C., Huang L., Zhang J., Zhang J., Zhu Y. (2018). Lewis Acid–Base Interaction-Induced Porous PbI2 Film for Efficient Planar Perovskite Solar Cells. ACS Appl. Energy Mater..

[28] Cao J., Wang F., Yu H., Zhou Y., Lu H., Zhao N., Wong C.-P. (2016). Porous PbI2 films for the fabrication of efficient, stable perovskite solar cells via sequential deposition. J. Mater. Chem. A.

[29] Jena A.K., Kulkarni A., Miyasaka T. (2019). Halide Perovskite Photovoltaics: Background, Status, and Future Prospects. Chem. Rev..

[30] Meng X., Chi K., Li Q., Feng B., Wang H., Gao T., Zhou P., Yang H., Fu W. (2021). Fabrication of Porous Lead Bromide Films by Introducing Indium Tribromide for Efficient Inorganic CsPbBr_3_ Perovskite Solar Cells. Nanomaterials.

[31] Kato Y., Ono L.K., Lee M.V., Wang S., Raga S.R., Qi Y. (2015). Silver Iodide Formation in Methyl Ammonium Lead Iodide Perovskite Solar Cells with Silver Top Electrodes. Adv. Mater. Interfaces.

[32] Kulkarni A., Ünlü F., Pant N., Kaur J., Bohr C., Jena A.K., Öz S., Yanagida M., Shirai Y., Ikegami M. (2021). Concerted Ion Migration and Diffusion-Induced Degradation in Lead-Free Ag_3_BiI_6_ Rudorffite Solar Cells under Ambient Conditions. Sol. RRL.

[33] Domanski K., Correa-Baena J.-P., Mine N., Nazeeruddin M.K., Abate A., Saliba M., Tress W., Hagfeldt A., Grätzel M. (2016). Not All That Glitters Is Gold: Metal-Migration-Induced Degradation in Perovskite Solar Cells. ACS Nano.

[34] Schlipf J., Docampo P., Schaffer C.J., Körstgens V., Bießmann L., Hanusch F., Giesbrecht N., Bernstorff S., Bein T., Müller-Buschbaum P. (2015). A Closer Look into Two-Step Perovskite Conversion with X-ray Scattering. J. Phys. Chem. Lett..

[35] Thompson C.V. (2012). Solid-State Dewetting of Thin Films. Annu. Rev. Mater. Res..

[36] Yang L., Wang J., Leung W.W.-F. (2015). Lead Iodide Thin Film Crystallization Control for High-Performance and Stable Solution-Processed Perovskite Solar Cells. ACS Appl. Mater. Interfaces.

[37] Liu X., Tan X., Liu Z., Ye H., Sun B., Shi T., Tang Z., Liao G. (2019). Boosting the efficiency of carbon-based planar CsPbBr_3_ perovskite solar cells by a modified multistep spin-coating technique and interface engineering. Nano Energy.

[38] Liu C., Li W., Li H.-Y., Wang H., Zhang C., Yang Y., Gao X., Xue Q., Yip H.-L., Fan J. (2019). Structurally Reconstructed CsPbI2 Br Perovskite for Highly Stable and Square-Centimeter All-Inorganic Perovskite Solar Cells. Adv. Energy Mater..

[39] Wang Z.-K., Li M., Yang Y., Hu Y., Ma H., Gao X.-Y., Liao L.-S. (2016). High Efficiency Pb-In Binary Metal Perovskite Solar Cells. Adv. Mater..

[40] Chen L., Wan L., Li X., Zhang W., Fu S., Wang Y., Li S., Wang H.-Q., Song W., Fang J. (2019). Inverted All-Inorganic CsPbI2Br Perovskite Solar Cells with Promoted Efficiency and Stability by Nickel Incorporation. Chem. Mater..

[41] Ko H.-S., Lee J.-W., Park N.-G. (2015). 15.76% efficiency perovskite solar cells prepared under high relative humidity: Importance of PbI2 morphology in two-step deposition of CH3NH3PbI3. J. Mater. Chem. A.

[42] Chen J., Zhao X., Kim S., Park N. (2019). Multifunctional Chemical Linker Imidazoleacetic Acid Hydrochloride for 21% Efficient and Stable Planar Perovskite Solar Cells. Adv. Mater..

[43] Yuan H., Zhao Y., Duan J., Wang Y., Yang X., Tang Q. (2018). All-inorganic CsPbBr_3_ perovskite solar cell with 10.26% efficiency by spectra engineering. J. Mater. Chem. A.

[44] Pham N.D., Tiong V.T., Chen P., Wang L., Wilson G.J., Bell J., Wang H. (2017). Enhanced perovskite electronic properties via a modified lead(ii) chloride Lewis acid–base adduct and their effect in high-efficiency perovskite solar cells. J. Mater. Chem. A.

[45] Ma J., Yang G., Qin M., Zheng X., Lei H., Chen C., Chen Z., Guo Y., Han H., Zhao X. (2017). MgO Nanoparticle Modified Anode for Highly Efficient SnO_2_-Based Planar Perovskite Solar Cells. Adv. Sci..

