# Supplementary material for: Controlled Growth of Porous InBr3: PbBr2 Film for Preparation of CsPbBr3 in Carbon-Based Planar Perovskite Solar Cells
Source: Nanomaterials (Basel). 2021 Sep 16;11(9):2408. doi: 10.3390/nano11092408 (PMC8465094; doi:10.3390/nano11092408)
Supplement: Supplementary file 1 [file nanomaterials-11-02408-s001.zip › nanomaterials-1362314-supplementary.pdf]

Supporting Information

Controlled Growth of Porous $InBr_3$: $PbBr_2$ Film for Preparation of $CsPbBr_3$ in Carbon-Based Planar Perovskite Solar Cells

Kailin Chi [1,*], Hansi Xu [1], Bingtao Feng [2], Xianwei Meng [2], Daoyu Yu [1] and Qian Li [3,*]

[1] School of Science, Northeast Electric Power University, Jilin 132012, China; 20182815@neepu.edu.cn (H.X.); 2018309050138@neepu.edu.cn (D.Y.)

[2] State Key Laboratory of Superhard Materials, College of Physics, Jilin University, Changchun 130012, China; fengbt20@mails.jlu.edu.cn (B.F.); xwmeng17@mails.jlu.edu.cn (X.M.)

[3] Beijing Key Lab of Cryo-Biomedical Engineering and Key Lab of Cryogenics, Technical Institute of Physics and Chemistry, Chinese Academy of Sciences, Beijing 100190, China; liqian@mail.ipc.ac.cn

* Correspondence: kailinchi@neepu.edu.cn (K.C.); liqian@mail.ipc.ac.cn (Q.L.); Tel./Fax: +86-0432-64806674 (K.C.); +86-010-82543763 (Q.L.)

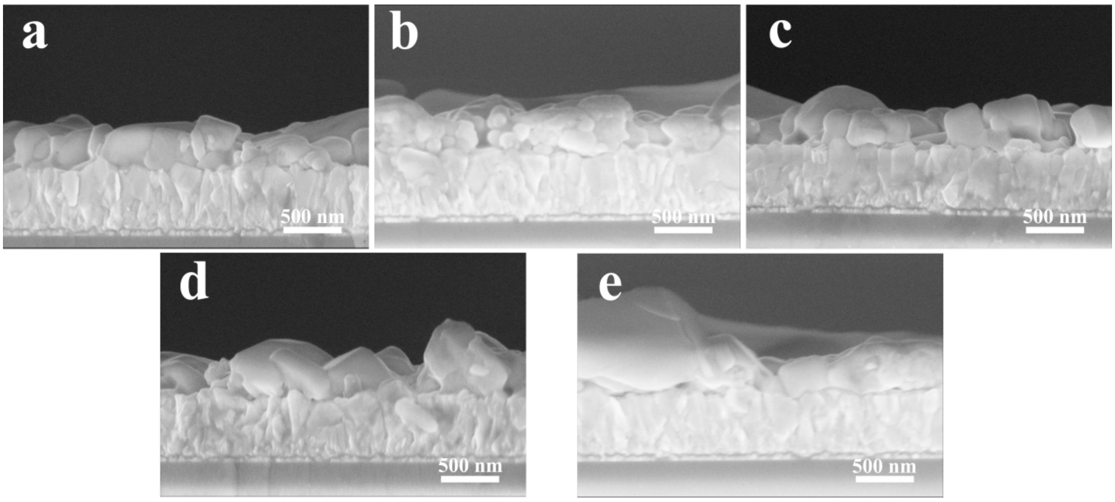

Figure S1. Cross-sectional SEM images of $PbBr_2$ films synthesized with different $PbBr_2$ concentration: (a) 1.0 M w/ $InBr_3$; (b) 1.1 M w/ $InBr_3$; (c) 1.2 M w/ $InBr_3$; (d) 1.3 M w/ $InBr_3$; (e) 1.4 M w/ $InBr_3$.

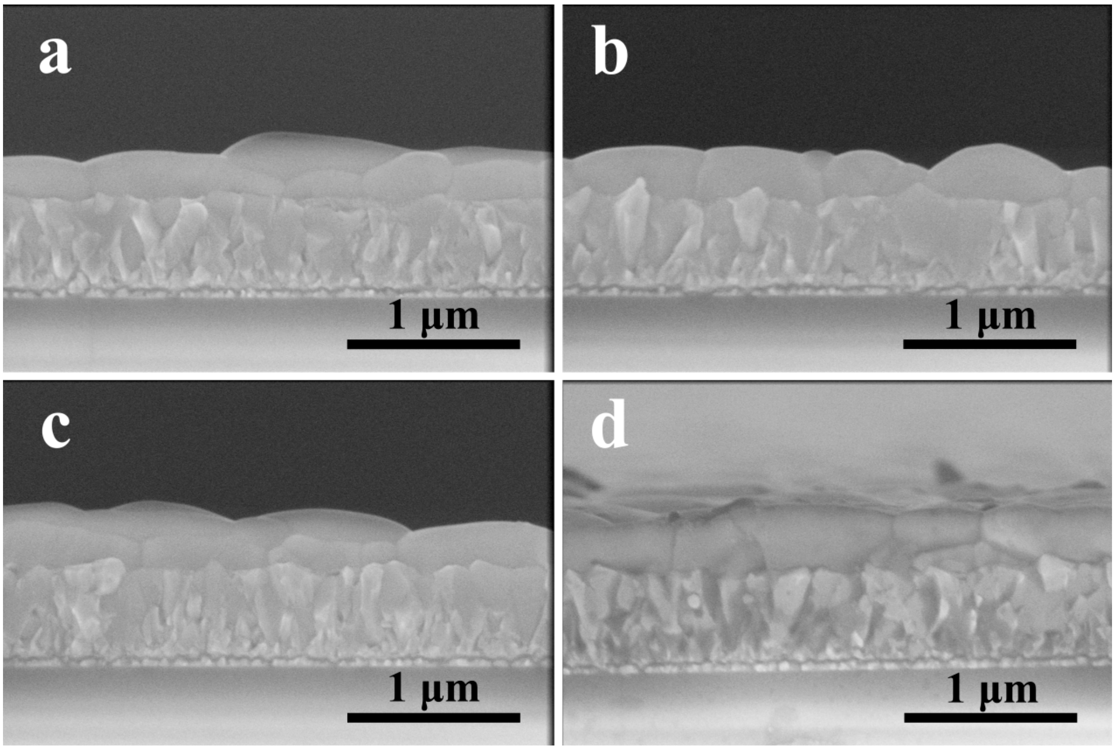

Figure S2. Cross-sectional SEM images of $CsPbBr_3$ films synthesized with different $PbBr_2$ concentration: (a) 1.1 M w/o $InBr_3$; (b) 1.2 M w/o $InBr_3$; (c) 1.3 M w/o $InBr_3$; (d) 1.4 M w/o $InBr_3$.

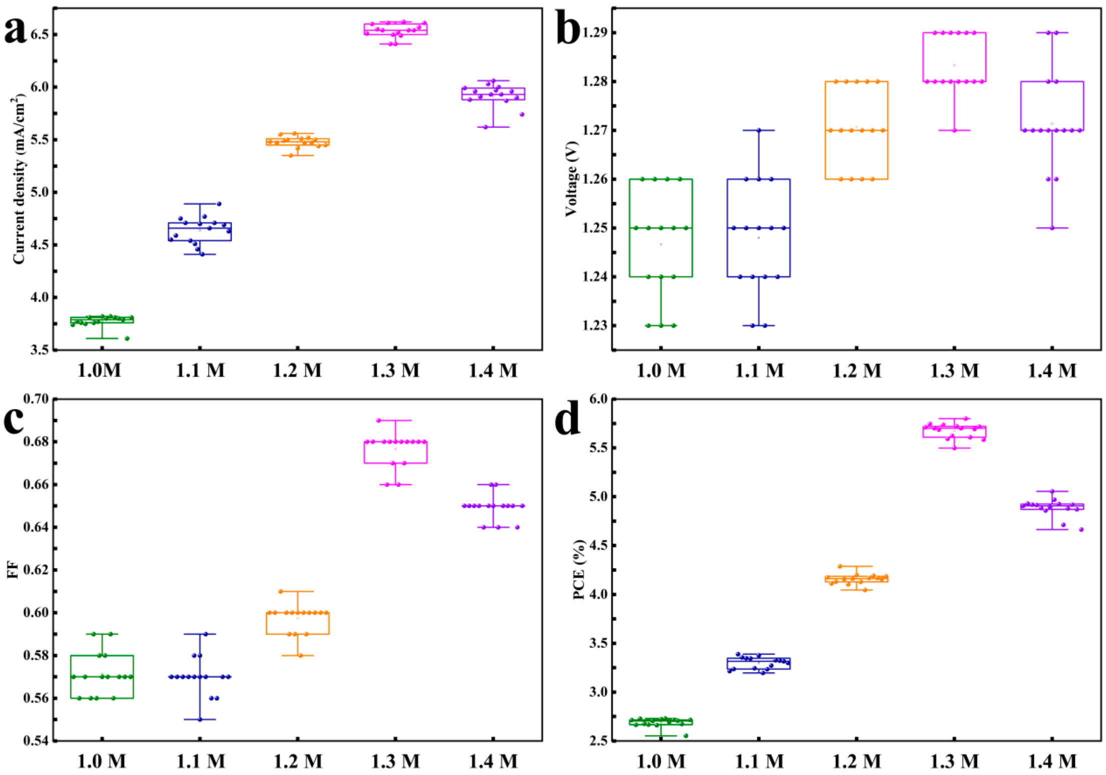

Figure S3. The schematic diagram of of 15 $CsPbBr_3$ PSCs synthesized with different $PbBr_2$ concentration: (a) J_{SC}, (b) V_{OC}, (c) FF and (e) PCE.

Table S1. The average photovoltaic parameters of 15 $CsPbBr_3$ PSCs synthesized with different $PbBr_2$ concentration.

$PbBr_2$ concentration	J_{SC} (mA/cm^2)	V_{OC} (V)	FF	PCE(%)
1.0 M w/$InBr_3$	3.77 ± 0.16	1.25 ± 0.02	0.57 ± 0.02	2.68 ± 0.14
1.1 M w/ $InBr_3$	4.64 ± 0.18	1.25 ± 0.02	0.57 ± 0.02	3.30 ± 0.13
1.2 M w/ $InBr_3$	5.48 ± 0.10	1.27 ± 0.01	0.60 ± 0.01	4.16 ± 0.13
1.3 M w/ $InBr_3$	6.54 ± 0.13	1.28 ± 0.01	0.68 ± 0.01	5.68 ± 0.18
1.4 M w/ $InBr_3$	5.92 ± 0.14	1.27 ± 0.02	0.68 ± 0.01	4.88 ± 0.12

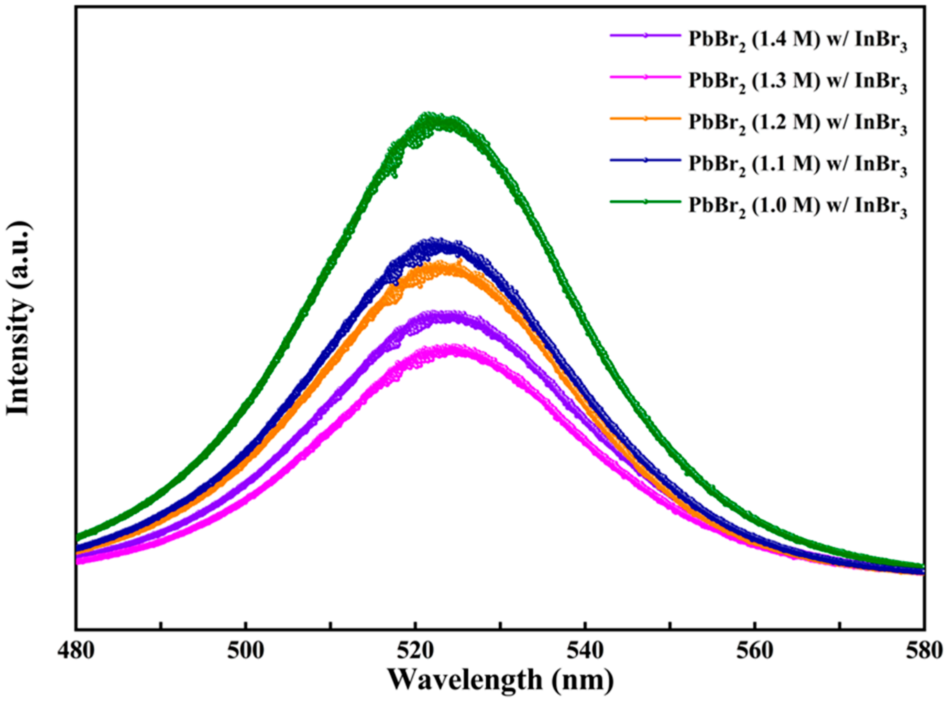

Figure S4. PL spectra of the $InBr_3$:$CsPbBr_3$ synthesized with different $PbBr_2$ concentration.

Table S2. The fitted data of TRPL characterization.

$CsPbBr_3$	τ_1 (ns)	A_1 (%)	τ_2 (ns)	A_2 (%)	τ_{ave} (ns)
**1.3 M w/ $InBr_3$**	**1.35**	**53.2**	**3.06**	**46.8**	**2.49**
**1.3 M w/o $InBr_3$**	**2.65**	**73.1**	**5.61**	**26.9**	**3.95**
